# Differential Roles for VviGST1, VviGST3, and VviGST4 in Proanthocyanidin and Anthocyanin Transport in *Vitis vinífera*

**DOI:** 10.3389/fpls.2016.01166

**Published:** 2016-08-03

**Authors:** Ricardo Pérez-Díaz, José Madrid-Espinoza, Josselyn Salinas-Cornejo, Enrique González-Villanueva, Simón Ruiz-Lara

**Affiliations:** Instituto de Ciencias Biológicas, Universidad de TalcaTalca, Chile

**Keywords:** anthocyanins, flavonoid transport, glutathione *S*-transferase, grapevine, GST, proanthocyanidins

## Abstract

In plant cells, flavonoids are synthesized in the cytosol and then are transported and accumulated in the vacuole. Glutathione *S*-transferase-mediated transport has been proposed as a mechanism involved in flavonoid transport, however, whether binding of flavonoids to glutathione *S*-transferase (GST) or their transport is glutathione-dependent is not well understood. Glutathione *S*-transferases from *Vitis vinífera* (VviGSTs) have been associated with the transport of anthocyanins, however, their ability to transport other flavonoids such as proanthocyanidins (PAs) has not been established. Following bioinformatics approaches, we analyzed the capability of VviGST1, VviGST3, VviGST4, and *Arabidopsis* TT19 to bind different flavonoids. Analyses of protein-ligand interactions indicate that these GSTs can bind glutathione and monomers of anthocyanin, PAs and flavonols. A total or partial overlap of the binding sites for glutathione and flavonoids was found in VviGST1, and a similar condition was observed in VviGST3 using anthocyanin and flavonols as ligands, whereas VviGST4 and TT19 have both sites for GSH and flavonoids separated. To validate the bioinformatics predictions, functional complementation assays using the *Arabidopsis tt19* mutant were performed. Overexpression of *VviGST3* in *tt19-1* specifically rescued the dark seed coat phenotype associated to correct PA transport, which correlated with higher binding affinity for PA precursors. *VviGST4*, originally characterized as an anthocyanin-related GST, complemented both the anthocyanin and PA deposition, resembling the function of *TT19*. By contrast, *VviGST1* only partially rescued the normal seed color. Furthermore the expression pattern of these *VviGSTs* showed that each of these genes could be associated with the accumulation of different flavonoids in specific tissues during grapevine fruit development. These results provide new insights into GST-mediated PA transport in grapevine and suggest that VviGSTs present different specificities for flavonoid ligands. In addition, our data provide evidence to suggest that GST-mediate flavonoid transport is glutathione-dependent.

## Introduction

Flavonoids are important plant secondary metabolites which include anthocyanins, flavonols, and proanthocyanidins (PAs, also named condensed tannins). They play important biological roles, including UV protection, reduction of reactive oxygen species (ROS) levels, defense against pathogens and herbivores as well as providing pigmentation to many flowers and fruits.

In plant cells, flavonoid biosynthesis can occur in different locations within the cytosol and then transported to be stored into vacuoles or other cellular compartments where they can fulfill biological functions. Because flavonoids are normally accumulated in cellular compartments different from those in which they are synthesized, their transport to the final site of storage is a critical process. To date, three distinct mechanisms for flavonoid transport have been proposed: vesicle trafficking, membrane transporters and glutathione *S*-transferase (GST)-mediated transport (Zhao, 2015). Small flavonoids-filled vesicles trafficking toward the main vacuole have been described (Poustka et al., 2007; Cutanda-Pérez et al., 2009; Conn et al., 2010; Gomez et al., 2011). In addition, a membrane trafficking factor (GFS9) was reported to be necessary for flavonoid accumulation in *Arabidopsis* vacuoles (Ichino et al., 2014). Regarding membrane transporters, members of the plant MATE family proteins have been implicated in flavonoid/H^+^ exchange (Baxter et al., 2005; Zhao et al., 2010). For instance, the *Arabidopsis* gene *TT12*, encoding a MATE protein, is required for PAs and anthocyanins accumulation in the vacuole (Debeaujon et al., 2001; Marinova et al., 2007). In the last years, orthologs of TT12 have been identified in other species, such as *Vitis vinifera* (Gomez et al., 2011; Pérez-Díaz et al., 2014) and *Medicago truncatula* (Zhao and Dixon, 2009; Zhao et al., 2011). ATP-binding cassette (ABC) transporters have also been involved in vacuolar sequestration of flavonoids. In maize, an ABCC-type transporter (MRP3) was implicated in anthocyanin transport (Goodman et al., 2004). More recently, Francisco et al. (2013) reported the participation of ABCC1 from *V. vinífera* in the transport of malvidin 3-*O*-glucoside into vacuoles. Anthocyanins and PAs are positively charged and therefore are not typical substrates for ABCC transporters, which catalyze the transfer of organic anions. However, animal ABCCs were reported to transport positively charged compounds in the presence of free glutathione (GSH; Konig et al., 2003). Because there is no evidence for the existence of glutathionylated anthocyanins (Zhao and Dixon, 2010), it is tempting to speculate that ABCCs mediate anthocyanin/GSH co-transport. In this regard, flavonoid transport mediated by Glutathione *S*-transferases could have a dual function, in the case they were associated to ABCC transporters (Zhao, 2015). Members of the multigenic GST family have been described to be essential for anthocyanin accumulation. Some examples are BZ2 in maize (Mueller and Walbot, 2001; Goodman et al., 2004); AN9 in petunia (Alfenito et al., 1998); Fl3 in carnation (Larsen et al., 2003) and TT19 in *Arabidopsis* (Kitamura et al., 2004, 2010). Kitamura et al. (2004) demonstrated that TT19 plays an additional role in deposition of PAs in seeds since a *tt19-1* mutant produced pale seed coats with a transparent testa phenotype due to a reduction in soluble PAs (Li et al., 2011). A novel GST from *Litchi chinensis* (LcGST4) was recently tested and proven to be the functional ortholog of the *Arabidopsis* TT19. However, this protein was related to the transport of anthocyanin and not of PAs. It was also shown that expression of *LcGST4* is activated by LcMYB1, a key regulator of anthocyanin synthesis in *Litchi* (Hu et al., 2016).

In grapevine, five GST genes have been studied in fruit (*VviGST1* to *VviGST5*; Conn et al., 2008). From all these, VviGST3 and VviGST4 present the highest homology to TT19 from *Arabidopsis*. Nonetheless, by performing complementation studies in the *bz2* maize mutant, Conn et al. (2008) demonstrated that only VviGST1 and VviGST4 are involved in anthocyanin accumulation in the vacuole. Furthermore, down-regulation of *VviGST4* expression in grapevine hairy roots revealed that this protein is necessary for anthocyanin transport from the ER to the tonoplast, independent of vesicle trafficking (Gomez et al., 2011). Taking into account that anthocyanins and PAs are the main flavonoids in grape fruits (Kennedy and Jones, 2001; Kennedy et al., 2001), the possibility that some of these GSTs may also have the ability to bind and transport PAs, cannot be discarded, because the model systems used to test the role of VviGSTs as flavonoid transporters (*bz2* kernels and grapevine hairy roots) do not accumulate PAs. On the other hand, none of the GSTs that mediate flavonoid transport may catalyze flavonoid glutathionylation and there is no direct evidence of flavonoids and GSH being co-mobilized by GSTs, since little is known about the interaction sites on the protein required for flavonoid binding.

To provide a better understanding on the GST-flavonoid and GST-GSH interactions and the participation of VviGST1, VviGST3, and VviGST4 in flavonoid transport, particularly PAs, we performed a molecular dynamics and docking analysis of the putative sites in GSTs for interaction with different flavonoid monomers and GSH. We also evaluated the expression pattern of these grapevine *GSTs* in different organs and during berry development and their ability to rescue the phenotype of the *Arabidopsis tt19-1* mutant. Our results suggest that these GSTs expressed during grapevine fruit development have different substrates specificities for three main types of flavonoids. In addition, our findings provide evidence to speculate that the GSTs require binding to GSH for efficient transport of flavonoids.

## Materials and Methods

### Plant Material and Growth Conditions

Plants of *V. vinifera* L. cultivar Carménère growing under field conditions in a commercial vineyard in the Maule Valley (Central Chile) during the 2012–2013 growing season were used in this study. Random sampling of different organs was performed including roots and leaves and fruits starting at early flowering until post-veraison (from November to March) from plants grown in the same plot. Stages to be sampled were defined according to the Modified Eichhorn-Lorenz System (Coombe, 1995): Flowers (EL 23), setting (EL 29), pre-veraison time 1 (EL 31), pre-veraison time 2 (EL33), veraison (EL 35), and post-veraison (EL 36). The skins and flesh samples were only readily separated from the rest of the berry from pre-veraison time 1. Leaves samples were collected at the stage of fully expanded mature leaf. All samples were frozen in liquid nitrogen and stored at -80°C until use for RNA isolation. Sampling was done at approximately 10:00 a.m. Wild-type Columbia-0, mutant *tt19-1* and transgenic *tt19-1* plants of *Arabidopsis thaliana* were grown in a mixture of vermiculite, perlite and peat moss (1:1:1) in a growth chamber at 23°C with a photoperiod of 16 h light and 8 h dark.

### RNA Isolation and cDNA Synthesis

The RNA purification from grapevine tissues was performed as described (Pérez-Díaz et al., 2014). All the RNA isolation for gene expression was done in triplicate for each tissue and developmental stage analyzed. Total RNA integrity was confirmed by formaldehyde agarose gel electrophoresis and the concentration and purity was determined with a NanoDrop ND-1000 Spectrophotometer (NanoDrop Technologies). RNA samples were treated with RNase free DNase I (Ambion) to remove contaminant DNA traces. To prepare first-strand cDNA, 2 μg of total RNA were reverse transcribed in a 20 μL reaction using the oligo d(T) and First Strand cDNA Synthesis Kit (Thermo Scientific) following the manufacturer’s instructions.

### Gene Expression Analysis

Gene transcript levels were measured by quantitative PCR (qPCR) using a Stratagene Mx3000P (Agilent Technologies) system. All reactions were performed using the Maxima SYBR Green/ROX qPCR Master Mix (Thermo Scientific) according to the manufacturer. For each sample (three biological replicates), qPCR was carried out in triplicate (technical repeats) using 10 μl Master Mix, 0.5 μl of 250 nM primers, 1 μl of diluted cDNA and nuclease-free water to a final volume of 20 μl. Amplification was followed by a melting curve analysis with continuous fluorescence acquisition during the 55–95°C melt. Raw data were manually analyzed and expression was normalized against *VvGAPDH* (accession number CN938023) and *VvUbiquitin1* (accession number TC32075) which present constant expression in vegetative and reproductive tissues (Almada et al., 2009; Gainza-Cortés et al., 2012; Pérez-Díaz et al., 2016). The primers used for qPCR analysis were: VviGST1-Fw, 5′-CCAAAGAGCAAAAGCCAAGT-3′ and VviGST1-Rv, 5′- TGTCCAGAAAACCCAAAGTC-3′ (Conn et al., 2008); VviGST3-Fw, 5′-TCCTGTCATTCAAGATGGAG-3′ and VviGST3-Rv, 5′-GGGTGGTAACTTTGTGCTTC-3′(Conn et al., 2008); VviGST4-Fw, 5′- CATACC AACAAGCCAACAAGCC-3′ and VviGST4-Rv, 5′-CTGAGAGGAAGAGTGTGA GTGC-3′; VvGAPDH-Fw, 5′-TTCCGTGTTCCTACTGTTG-3′ and VvGAPDH-Rv, 5′-CCTCTGACTCCTCCTTGAT-3′; VvUbiquitin1-Fw, 5′-GTGGTATTA TTGAGCCATCCTT-3′ and VvUbiquitin1-Rv, 5′-AACCT CCAATCCAGTCATCTAC-3′ (Downey et al., 2003a).

### Genetic Construction and Plant Transformation

The coding sequences of *VviGST1, VviGST3*, and *VviGST4* were amplified from cDNA of berry at veraison stage using the following primers: VviGST1-Fw, 5′-GGATCCATGGCAAAC AGTGACCACAT-3′ and VviGST1-Rv, 5′-GAGCTCTCAAACA ACCGCAATAATAT-3′; VviGST3-Fw, 5′-CCCGGGATGGTGG TGAAGGTGTATG-3′ and VviGST3-Rv, 5′-GAGCTCTCACTC CAAGAGGGGCCAT-3′; VviGST4-Fw, 5′- GGATCCATGGTG ATGAAGGTGTATGGCCCA-3′ and VviGST4-Rv, 5′-GAGCT CTCAAGCAGCGAGCTCCATGACTTTT-3′. The amplification reactions were carried out with the Platinum^®^ Taq DNA Polymerase (Invitrogen) and the resulting DNA fragments were cloned into pGEM-T vector (Promega) and sequenced. The PCR products were inserted in the sense orientation into the SmaI-SacI (*VviGST1*), XbaI-SacI (*VviGST3*), and BamHI-SacI (*VviGST4*) sites of the pBI121 binary vector to replace the β- *glucuronidase* (*GUS*) gene, resulting in the *VviGSTs* genes being under the control of the CaMV 35S promoter. The final expression vectors were introduced into *Agrobacterium tumefaciens* strain GV3101. Transformations of the mutant line *tt19-1* of *A. thaliana* were performed using the floral-dip method as reported by Clough and Bent (1998). Transgenic plants were selected on MS medium containing 50 mg L^-1^ kanamycin and 500 mg L^-1^ augmentin. The generated kanamycin-resistant seedlings were then transferred to a substrate mixture and grown as indicated earlier. The presence of the transgene was confirmed by PCR from gDNA using specific primers for *VviGST* genes (listed above). Isolation of genomic DNA was performed using the Wizard^®^ Genomic DNA Purification Kit (Promega). T2 plants were used to perform phenotypic analysis.

### Phylogenetic Analysis and Multiple Alignment

The full-length amino acid sequences of VviGST1, VviGST3, VviGST1, and other plant GST proteins were used as templates to perform a multiple-alignment using the BioEdit Sequence Alignment Editor v7.0 software (Hall, 1999). A phylogenetic tree was built from the resulting aligned sequences using the MEGA software^1^ (version 4; Tamura et al., 2007) and the neighbor-joining method with bootstrap analysis of 1,000 replicates. The GenBank accession numbers of plant GST proteins used for alignment and phylogenetic analysis are the following: *V. vinifera* VviGST1 (AAN85826), VviGST3 (ABO64930), VviGST4 (NP_001267869), VviGST5 (ABL84692); *A. thaliana* TT19/AtGSTF12 (AED92398); *Petunia hybrid*a AN9 (CAA68993); *L. chinensis* LcGST4 (ALY05893); *Cyclamen* sp. CkmGST3 (BAM14584); *Perilla frutescens* PfGST1 (BAG14300); *Zea mays* BZ2 (AAA50245); *Glycine max* GmGST26A (NP_001238439).

### Structure Prediction by Homology Modeling

The structural models of VviGST1, VviGST3, VviGST4, and TT19 were built by homology modeling based on crystal structures of homologous proteins. SWISS-MODEL (Biasini et al., 2014) was used to select 3D models crystallized with the closest sequence homology and also to construct comparative model structures. The model with the best homology to GST3, GST4, and TT19 was the crystalline structure of a Phi class GST from *Populus tremula* × *P. tremuloide* PttGSTF1 (PDB code 4RI6; Pégeot et al., 2014) and for GST1 was the structure of a Tau class GST from *G. max* GmGSTU10-10 (PDB code 5AGY; Axarli et al., 2016). The best models for GST proteins obtained by SWISS-MODEL were improved by molecular dynamic simulation and equilibration methods using Nano Molecular Dynamics (NAMD v. 2.10; Phillips et al., 2005), the Chemistry of Harvard Molecular Modeling (CHARMM27) force field (Schlenkrich et al., 1996) and the TIP3P model for water (Jorgensen et al., 1983). A short initial minimization of 15,000 steps was used to remove wrong contacts and for energy optimization. The molecular dynamics were done using the following conditions: 12 ns of molecular dynamics, a periodic bordering condition box (80 Å × 100 Å × 80 Å), 150 mM NaCl and 300°K with default parameters (Phillips et al., 2005). The final 3D model of each structure was evaluated for its stereochemical quality and atomic coordinates with a Ramachandran map using PROCHECK (Laskowski et al., 1993) and the ProSA program (Sippl, 1993), respectively.

### Molecular Dynamics and Docking Studies

All docking studies described here involved flexible docking of the ligand to the rigid receptor. Ligand sequences were searched using the PubChem Project Server. Each ligand [Cyanidin 3-*O*-glucoside (PubChem code 197081), (-)-Epicatechin-3-*O*-gallate (PubChem code 107905), Kaempferol 3,7-di-O-α-rhamnopyranoside (PubChem code 12305419), Quercetin-3-*O*-rhamnoside (PubChem code 15939939) and GSH (PubChem code 124886)] and protein in the dataset was processed as following. Input structures for the ligands were prepared by using the MOPAC2012 which includes the method of PM6 charges (Stewart, 2012). For protein structures, water molecules were removed and hydrogen atoms were added. The partial charges of the atoms were assigned using the Maestro software (Schrödinger, LLC, New York, NY, USA, 2015 release). The AutoDockTools 1.4.6 software (ADT; Morris et al., 2009) was used for docking studies. ADT was used for establishing the Autogrid points as well as visualization of docked protein-ligand complex structures. Grid parameter files were built and atom-specific affinity maps were constructed using Autogrid 4 (Huey et al., 2007). These map files were generated using 100 × 100 × 100 grid points and 0.375 Å spacing, with the maps centered on the determined H-site of each protein. Docking simulations for the study were carried out using the Lamarckian Genetic Algorithm of Autodock 4. The initial position, orientation and torsions of the ligand molecules were set randomly, and all rotatable torsions were released during docking. Each docking experiment was derived from 100 runs that were set to terminate after a maximum of 2,500,000 energy evaluations. The Python Molecular Viewer 1.4.5 (Sanner, 1999) and the visual molecular dynamics (VMD; Humphrey et al., 1996) softwares were used for final visualizations.

### Determination of Anthocyanin Content

Total anthocyanin content was determined in *Arabidopsis* seedlings from wild-type and transgenic lines using a spectrophotometric differential pH method, according to Vankar and Srivastava (2010). The results were expressed as micrograms of cyanidin-3-glucoside equivalents per gram of fresh weight.

### DMACA Staining

*Arabidopsis* seeds of Columbia-0 wild-type, *tt19-1* and *tt19-1* overexpressing *VviGST1*, *VviGST3*, and *VviGST4* were stained with dimethylaminocinnamaldehyde (DMACA) reagent following the protocol reported by Li et al. (1999).

## Results

### Three-Dimensional Structures of VviGSTs Reveal Binding Sites for Flavonoids

The ability of VviGST to bind flavonoids was studied using bioinformatics analyses. For this, two *VviGSTs* genes (*VviGST3* and *VviGST4*) that encode proteins with high homology to TT19 (over 46% of identity) and *VviGST1* encoding a protein homolog to BRONZE2 (BZ2) from maize were selected. The full-length coding sequences of these three genes were amplified and cloned and their deduced encoded proteins were identical to their previously described sequences (Conn et al., 2008). Phylogenetic analysis shows that VviGST3 and VviGST4 are grouped in the same clade with *Arabidopsis* TT19 and other GSTs from the group Phi previously characterized as flavonoid transporters such as AN9 (Mueller et al., 2000) and PfGST1 (Yamazaki et al., 2008; **Supplementary Figure S1**). In contrast, other proteins involved in anthocyanin transport such as maize BZ2 (belonging to group Tau of GSTs) and VviGST1 grouped separately. Comparison of the deduced amino acid sequences of VviGSTs and other GSTs involved in flavonoid transport indicate the presence of residues described as important for binding to GSH (Conn et al., 2008; **Supplementary Figure S2**). To test whether VviGST1, VviGST3, and VviGST4 can dock flavonoids, we used a structural approach considering the capability of TT19 to bind anthocyanins and PAs. First, three-dimensional models of TT19, VviGST3, and VviGST4 were constructed using the crystal structure of the poplar PttGSTF1 (PDB code 4RI6) Phi class GST protein template, and the model of VviGST1 using the structure of the *G. max* GmGSTU10-10 (PDB code 5AGY) Tau class GST. The PttGSTF1 protein has 43.61% sequence identity with TT19, 49.76% with VviGST3 and 49.50% with VviGST4, while GmGSTU10-10 has 60% identity with VviGST1. The refined structural models of TT19, VviGST1, VviGST3, and VviGST4 were obtained (**Supplementary Figure S3**). All models presented high conformational quality according to Ramachandran analysis (**Supplementary Figure S4**). 100% of the amino acids of VviGST4 and 99.5% of TT19, VviGST1, and VviGST3 were spatially well distributed, illustrating the stereochemical quality of the models. In addition, the ProSA analysis confirmed that the models have energy qualities comparable to a reference template (**Supplementary Figure S5**).

All models displayed similar 3D structures, with N-terminal domains having a mixture of small β-sheets and α-helices and with C-terminal domains formed exclusively of α-helices. The putative binding site for GSH (named G-site) is located in the N-terminal domain of each protein (Liu et al., 2013) and its architecture appears to be similar among TT19, VviGST3, and VviGST4, while that in VviGST1 has a different orientation (**Supplementary Figure S3**). The amino acid composition of the site is highly conserved and is defined by the residues Lys40, Pro49, Gln52, Glu65, Ser66, Tyr105, Gln112, and Lys139 in TT19 (**Supplementary Figure S2**; Reinemer et al., 1996). The putative binding site for flavonoids (named H-site) is also located in the N-terminal domain (Pégeot et al., 2014) and does not differ significantly in spatial structure among the different GSTs.

After refined structural models were obtained for each of the GSTs, an automated docking was used to assess the binding free energy (ΔG) of the protein-GSH and protein-ligand interactions with different flavonoids [Cyanidin-3-*O*-glucoside (Cy3G), (-)-Epicatechin-3-*O*-gallate (Ep3G), Kaempferol 3,7-di-*O*-α-rhamnopyranoside (Ka3,7R) and Quercetin-3-*O*-rhamnoside (Qu3R)]. We calculated the ΔG for the PttGSTF1-GSH and GmGSTU10-10-GSH interactions in order to get reference values to compare with those obtained for VviGSTs-ligand interactions (**Table 1**). The ΔG values for the five ligands were negative with all GSTs, indicating that they can bind these substrates (**Table 1**). The values obtained with TT19 and ligands Ep3G and Cy3G (-2.43 and -2.49 K cal mol^-1^) seem to confirm the ability of this GST to bind and transport anthocyanins and PAs. Besides it serves as a reference to suggest that the three GSTs from grapevine also have the ability to bind Ep3G and Cy3G. The values also suggest that VviGST1 and VviGST3 have higher affinity for PAs than anthocyanins. In contrast TT19 and VviGST4 have similar affinities for both types of flavonoids (**Table 1**). Interestingly, the ΔG of the flavonol Qu3R was very high for all GSTs, suggesting that these proteins could also be involved in the transport of alternative ligands of flavonoid origin.

**Table 1:** Prediction of the protein binding affinity between flavonoid with H-site and glutathione with G-site in GSTs protein.

Ligand	ΔG (Kcal/mol)			
	TT19	VviGSTl	VviGST3	VviGST4
Cy3G	-2,43	-2,77	-2,03	-2,2
Ep3G	-2,49	-3,69	-4,57	-2,67
Qu3R	-3,90	-4,77	-3,39	-4,70
Ka3,7R	-2,11	-2,38	-2,84	-1,37
GSH	-1,56	-1,08	-1,67	-2,70

*Cy3G means Cyanidin-*O*-3-Glucoside; Ep3G, (-)-Epicatechin-3-0-Gallate; Qu3R, Quercetin-3-*O*-Rhamnoside; Ka3,7R, Kaempferol-3,7-di-0-a-rhamnopyranoside; GSH, Glutathione; ΔG, Gibbs free energy. Internal controls: interaction energy of PUGSTF1 - GSH: -2,08 Kcal/mol and GmGSTU10-10 -GSH: -2,67 Kcal/mol.*

**FIGURE 1:**
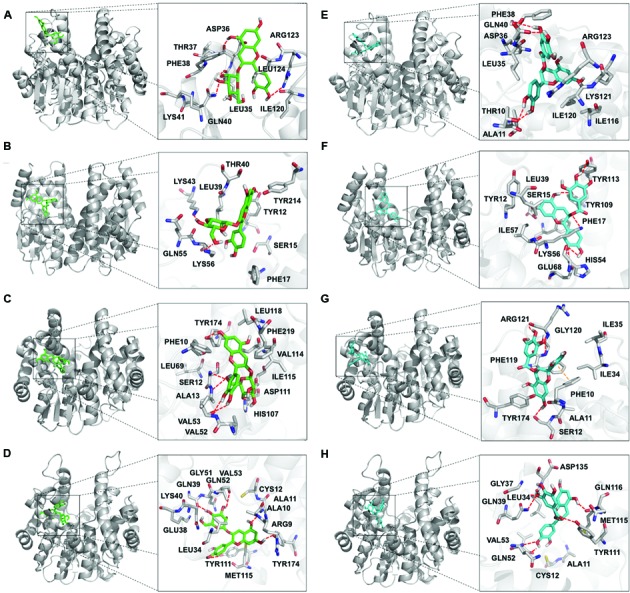
**Diagram of the interactions of the flavonoid ligands at the binding site in glutathione *S*-transferase (GSTs).** GSTs protein or peptide bonds that interact with the ligands are shown in gray. **(A,E)** TT19. **(B,F)** VviGST1. **(C,G)** VviGST3. **(D,H)** VviGST4. **(A–D)** Cyanidin 3-*O*-glucoside in green. **(E–H)** (–)-Epicatechin-3-*O*-gallate appear in cyan. H-bond interactions are in red and π–π interactions in orange.

According to the ΔG for protein-ligand interactions, the putative amino acids involved in the binding were determined. In the TT19-Cy3G interaction, the residues that participate are: Leu35, Asp36, Gln40, and Arg123 (through hydrogen bonding [H-bonding]); Phe17, Leu39, and Tyr214 (by hydrophobic interactions [HI]); and Lys41 and Thr37 (participating in the conformational structure of the H-site [SH]) (**Figure 1A**). In the VviGST1-Cy3G interaction, the amino acids are: Tyr12, Lys43, and Lys56 (H-bonding); Phe17, Leu39 and Tyr214 (HI) and Ser15, Thr40 and Gln55 (SH) that are in the cavity of the H-site (**Figure 1B**). In VviGST3-Cy3G, the residues that contribute to the interaction are: Ser12, Ala13, Val53, and Tyr154 (H-bonding); Phe10, Val52, Val114, Ile115, Leu118, and Phe219 (HI) and Leu69, His107 and Asp111 (SH) that are located at the H-site (**Figure 1C**). For the VviGST4-Cy3G interaction, the amino acids are: Ala11, Gln39, Val53, Tyr111, and Tyr174 (H-bonding); Ala10, Cys12, Leu34 and Met115 (HI) and Arg9, Glu38, Lys40, Gln51 and Gln52 (SH) that are present at the H-site (**Figure 1D**). Of these interactions, the residues Leu35 and Gln40 were found frequently in these GSTs and other anthocyanin-related GSTs (**Supplementary Figure S2**; Kitamura et al., 2012). In the case of TT19-Ep3G, the amino acids that contribute to the interaction are: Thr10 and Gln40 through H-bonding; Ala11, Leu35, Phe38, Ile116, Ile120, and Leu124 with HI and Lys41, Lys121 and Arg123 (SH) that also are present at the H-site (**Figure 1E**). For VviGST1-Ep3G, the residues are: Lys56, Glu68, Tyr109 and Tyr113 (through H-bonding); Tyr12, Phe17, Leu39 and Ile57 (by HI) and Ser15 and His54 that also are part of the H-site (**Figure 1F**). In the case of VviGST3-Ep3G, the amino acids that participate in the interaction are: Ser12, Gly120 and Arg121 (H-bonds); Phe10 participate through a π–π interaction and Ala11, Ile34, Ile35, Tyr174, and Phe119 by HI (**Figure 1G**). Finally, the residues that contribute in the VviGST4-Ep3G interaction are: Leu34, Aps35, Gln39, Val53, Tyr111, and Met115 (H-bonding); Ala11, Cys12 and Pro13 (HI) and Gly37, Gln52 and Gln116 that are present at the H-site (**Figure 1H**). Of these analyses, the residue Ala11 was present in all the phi-class GSTs and this would be relevant for the interactions with Ep3G. Moreover, the amino acid Leu35 was often present again in interactions with Ep3G, revealing its importance to the binding with anthocyanin and PAs.

**FIGURE 2:**
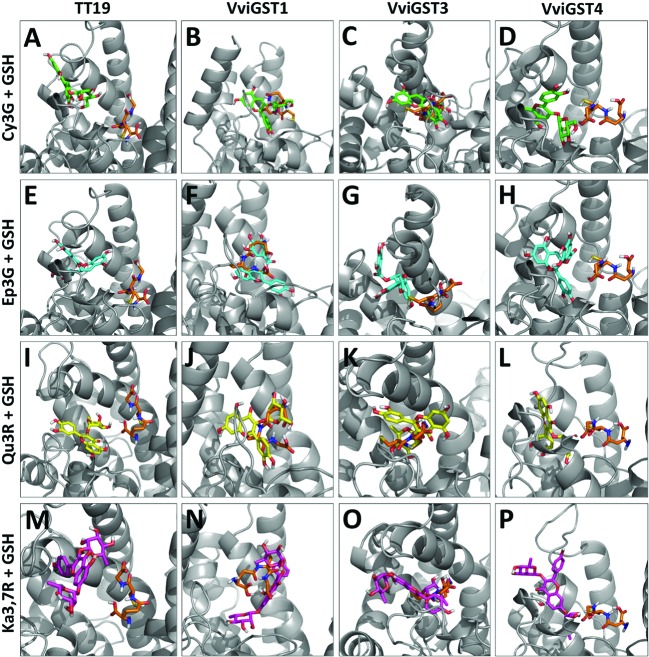
**Diagram of flavonoid versus GSH in energetically favorable docking sites. (A–D)** Cy3O (Cyanidin 3-*O*-glucoside) in green. **(E–H)** Ep3G [(–)-Epicatechin-3-*O*-gallate] in light blue. **(I–L)** Qu3R (Quercetin-3-*O*-rhamnoside) in yellow. **(M–P)** Ka3,7R (Kaempferol 3,7-di-*O*-α-rhamnopyranoside) in pink. In all panels, glutathione appears in orange and GST proteins in gray.

The GST proteins analyzed show a favorable ΔG to GSH on the G-site, but lower that for flavonoid ligands at H-site, except for VviGST4 (**Table 1**). Because the close location of the G-site and H-site, we overlaid the models that show the best ΔG with their ligands in order to determinate whether both ligands prefer the same binding site (**Figures 2A–P**). The ligand Cy3G in TT19 and VviGST4 or Ep3G in TT19, VviGST3, and VviGST4 did not appear to use the same binding site than GSH (**Figures 2A–H**). In contrast, these same ligands have preference for the same binding site that GSH has in VviGST1 (**Figures 2B,F**). Also, Cy3G seems to prefer the GSH binding site in VviGST3 (**Figure 2C**). In addition, TT19 and VviGST4 show different binding sites for GSH and Ka3,7R or Qu3R (**Figures 2I,L,M,P**). By contrast, these flavonols use the same binding site that GSH uses in VviGST1 and VviGST3 (**Figures 2J,K,N,O**).

### *VviGST1*, *VviGST3*, and *VviGST4* Are Differentially Expressed during Grape Berry Development

**FIGURE 3:**
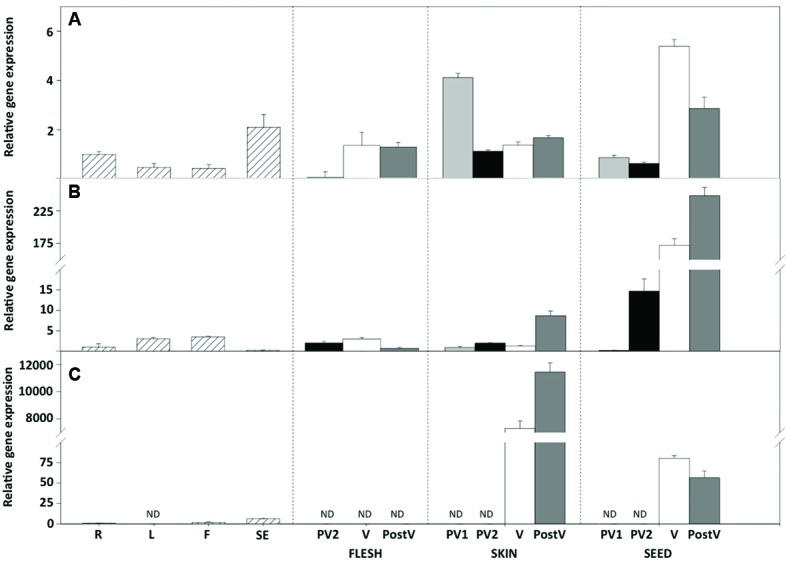
**Gene expression profiles of *VviGST1*, *VviGST3*, and *VviGST4* in vegetative and reproductive tissues of *Vitis vinifera* cv Carménère.** Expression analysis of **(A)** *VviGST1*, **(B)** *VviGST3*. **(C)** *VviGST4* in roots (R); leaves (L); flowers (F); fruit setting (SE); pre veraison time 1 (PV1); pre veraison time 2 (PV2); veraison (V); and postveraison (PostV). Berries were dissected in skins (black bars), seeds (light gray bars) and flesh (gray bars). Gene expression was evaluated by real time quantitative PCR and normalized against expression of *VvGAPDH*. Data points represent means of three biological and three technical replicates ± SD. ND, not detected.

Since flavonoids are differentially accumulated in berry tissues, transcriptional analysis of the *VviGST* genes was carry out in skins, seeds and fruit flesh through different developmental stages. Spatial and temporal gene expression analysis of *VviGST3* revealed that its transcripts were almost exclusively accumulated in seeds during fruit development compared with the rest of the tissues, with an increase at veraison and the maximum level detected at post-veraison stage (**Figure 3**). In contrast, its transcript levels were less abundant in grape skins and flesh, similar to those found in other vegetative organs (**Figure 3**). To compare these findings, we also analyzed the expression profile of anthocyanin-related *GST* genes previously reported (Conn et al., 2008). *VviGST4* was highly and almost exclusively expressed in berry skins from the veraison stage, with a peak of transcripts detected at post-veraison (**Figure 3**). This expression pattern correlates with anthocyanin synthesis in grape fruit and is consistent with its role as anthocyanin transporter as previously demonstrated in grapevine plants (Gomez et al., 2011). In the case of *VviGST1*, its transcripts were differentially accumulated in both berry skins and seeds, with the highest transcript levels observed in seeds from veraison until post-veraison (**Figure 3**). Interestingly, at the early stage of pre-veraison, *VviGST1* showed the highest expression in skins than seeds. On the other hand, *VviGST1* expression was weak in flesh and other vegetative tissues. The expression pattern of *VviGST1* suggests its participation during early development (fruit setting) and skins and a function in seeds during fruit ripening. In summary, these results indicate a different organ/tissue and temporal regulation of *VviGSTs* during grapevine fruit development, which could be related to the synthesis and accumulation of different types of flavonoids.

### Grapevine *GSTs* Showed Different Capabilities to Restore Anthocyanin and PA Transport Deficiency of the *Arabidopsis tt19-1* Mutant

**FIGURE 4:**
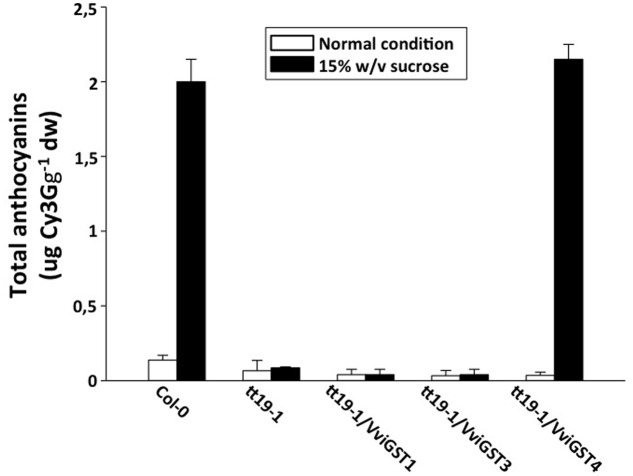
**Total anthocyanins content in *Arabidopsis* leaves of wild-type, *tt19-1* and transgenic plants expressing *VviGST1*, *VviGST3*, and *VviGST4*.** Values represent means of three replicates ± SD. Cy3G: Cyanidin-3-glucoside. White bars indicate normal conditions and black bars indicate anthocyanin-induced condition [15% (w/v) sucrose].

**FIGURE 5:**
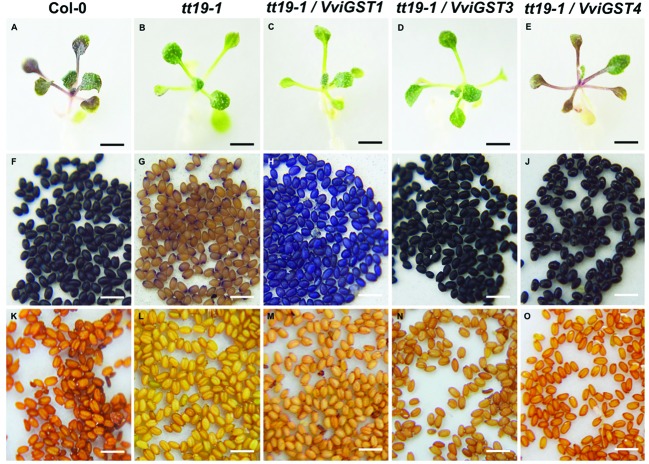
**Evaluation of the phenotypic complementation of the *tt19-1* mutant expressing *VviGST1*, *VviGST3* and *VviGST4*. (A,F,K)** Col-0. **(B,G,L)** *tt19-1*. **(C,H,M)** *VviGST1*. **(D,I,N)** *VviGST3*. **(E,J,O)** *VviGST4*. **(A–E)** 25-day seedlings were grown on agar plates in 15% (w/v) sucrose to promote anthocyanin accumulation. **(F–J)** Seeds stained with DMACA. **(K–O)** Unstained seeds. Black bar = 5 mm. White bar = 1 mm.

To investigate the possible functions of *VviGSTs* in flavonoid transport *in planta*, we performed a functional complementation assay. It has been reported that the *Arabidopsis tt19-1* mutant has a defect in anthocyanin accumulation in vegetative tissues and a pale seed testa due to a failure in anthocyanin and PAs transport and accumulation. Thus, transgenic *tt19-1* plants expressing *VviGST3*, *VviGST1*, and *VviGST4* under the control of the CaMV 35S promoter were generated and their transparent testa phenotype was evaluated. Constitutive expression of *VviGSTs* genes showed different capabilities to restore the normal transport of flavonoids in the *tt19-1* mutant. In a treatment with sucrose to induce anthocyanin accumulation, wild-type plants rapidly accumulated anthocyanins in their leaves (**Figures 4** and **5A**), nevertheless the lack of *TT19* transcript in the mutant abrogated anthocyanin accumulation in their leaves under sucrose stress (**Figures 4** and **5B**). Transgenic *tt19-1* plants expressing *VviGST3* were unable to accumulate anthocyanins as wild-type plants did (**Figures 4** and **5D**). However, when we analyzed accumulation of PAs (as well as their immediate precursor molecules) in the seed coats using DMACA staining, a complete recovery of the dark seed color was observed, which was similar to wild-type seeds but darker compared to the pale mutant seeds (**Figures 5F,G,I,K,L,N**). This result is consistent with the high expression of *VviGST3* in berry seeds (that accumulate large amounts of PAs) and suggest that VviGST3 may function in a similar way than TT19 in *Arabidopsis* seeds in terms of its affinity for the PA precursor Ep3G (**Table 1**). *VviGST1* and *VviGST4* have been shown to transport anthocyanin when expressed transiently in maize *bz2* mutant lacking a GST involved in flavonoid transport (Conn et al., 2008). Further functional characterization of *VviGST4* in transgenic grapevine demonstrated its role as an anthocyanin transporter (Gomez et al., 2011). As far as we know, neither VviGST4 nor VviGST1 have been evaluated in their ability to bind and transport other flavonoids present in grape fruit such as PAs. As expected, expression of *VviGST4* in *tt19-1* mutant plants recovered the phenotype of anthocyanin accumulation (**Figures 4** and **5E**), but most interestingly, recovered the normal dark seed coloration (**Figures 5J,O**). This finding shows that VviGST4 can also bind PAs and participate in their transport, resembling, in part, the function of TT19 in *Arabidopsis* and is in agreement with our bioinformatics predictions (**Table 1**). Transgenic *tt19-1* lines overexpressing *VviGST1* were not able to recover the anthocyanin-less phenotype in seedlings exposed to sucrose stress (**Figures 4** and **5C**). In contrast, histochemical staining of PAs in their seeds revealed a partial complementation of the transparent testa phenotype when compared to seeds of the *tt19-1* mutant (**Figures 5H,M**).

Altogether, our data indicate that different *VviGSTs* expressed in grape fruits have the ability to bind flavonoid ligands with different specificities and suggest that the transport of these ligands into their cellular compartment may be dependent on the binding of GSH to the GSTs.

## Discussion

In recent years, significant progress has been made to better understand flavonoid transport mechanisms in plants (Zhao and Dixon, 2010; Zhao, 2015). Among these mechanisms, the transport mediated by glutathione *S*-transferases (GSTs) has been focused on anthocyanin transport but evidence of their involvement in the transport of other flavonoids such as PAs and flavonols has been limited. GSTs belong to a multigene family in plants, i.e., 55 GSTs in *Arabidopsis* (Dixon and Edwards, 2010), 59 GSTs in rice (Soranzo et al., 2004) and at least 87 members have been predicted in *V. vinífera* (Ali et al., 2011). These proteins have been classified in subclasses Phi, Zeta, Theta, Tau, DHAR, and Lambda, where Phi and Tau GSTs are exclusive to plants. Plant GSTs are considered enzymes of the secondary metabolism and are typically associated with xenobiotic detoxification, whereas other basic functions are much less understood. Therefore, the diversity of functions of these proteins can involve GSH-dependent and GSH-independent activities and binding functions (Dixon et al., 2010). GST’s ligand activity has been considered of interest since it may contribute to intracellular transport or sequestration of a diversity of molecules such as flavonoids (Falcone-Ferreyra et al., 2012).

In grapevine, anthocyanins and PAs are the main flavonoids in fruits, which are associated to organoleptic properties in red wine such as color, bitterness, and astringency. Despite this, there is no experimental evidence linking grapevine GSTs with the binding and transport of PAs or other flavonoids other than anthocyanins (Conn et al., 2008; Gomez et al., 2011). Out of the GSTs analyzed in grape fruit, only VviGST4 and VviGST1 have been described to be related to anthocyanin transport. To date, the putative amino acids involved in the interaction between GSTs and flavonoids has been proposed based only in structural approximations performed with prokaryotic GST (Conn et al., 2008) or lineal approximations of anthocyanin-related GSTs (Kitamura et al., 2012) but without performing molecular docking analysis (**Supplementary Figure S6**). In this work, we selected VviGST1, VviGST4, and VviGST3, a protein highly homologous to *Arabidopsis* TT19 and VviGST4, to study their role in the flavonoid transport. Applying bioinformatics approaches we built structural models for VviGST1, VviGST3, VviGST4, and *Arabidopsis* TT19 (**Supplementary Figure S3**) and the binding free energies of the interaction flavonoid-GST and GSH-GST were calculated using molecular docking (**Table 1**). Anthocyanins, flavonols and PAs are differentially accumulated in various organs/tissues during fruit development of grapevine (Kennedy et al., 2001; Downey et al., 2003a,b; Bogs et al., 2005; Verries et al., 2008). The expression patterns of *VviGSTs* were analyzed in detail in fruit tissues at different developmental stages, in order to correlate the gene expression of *GSTs* with the flavonoids transport. To validate the bioinformatics results, we perform a functional complementation assay with the *Arabidopsis tt19-1* mutant because this model present advantages over other mutants, such as *bz2* from maize (defective in the anthocyanin transport) to study of different flavonoid-related *VviGST* candidates (Marrs et al., 1995; Alfenito et al., 1998; Conn et al., 2008; Yamazaki et al., 2008; Kitamura et al., 2010; Hu et al., 2016).

VviGST1, a Tau subclass GST related to anthocyanin transport and homolog to BZ2 (**Supplementary Figure S1**; Conn et al., 2008), showed to be able to bind the four types of common flavonoids. However, all those interactions showed to be partly or completely overlapping to GSH binding site (**Figure 2**). The ΔG values were more favorable to Qu3R and Ep3G than to Cy3G, suggesting that VviGST1 could have a predominant role in the transport of other flavonoids different from anthocyanin. Despite that, *VviGST1* was originally suggested to participate in anthocyanin transport based on its capability to complement *bz2* mutant kernels (Conn et al., 2008), its expression pattern may not be easily associated with the profile of anthocyanin accumulation in fruits and rather seems to correlate with the accumulation of flavonols and PAs (**Figure 3**). In this respect, our results do not agree with those reported by Conn et al. (2008), since *tt19-1* mutant plants overexpressing *VviGST1* were not able to accumulate anthocyanins in their leaves upon sucrose treatment (**Figures 4** and **5C**). The discrepancy in these results may be due to the high structural homology between VviGST1 and BZ2 or that available anthocyanins in *Arabidopsis* leaves are different from those present in maize seeds. On the other hand, *VviGST1* partially rescued the phenotype of *tt19-1* suggesting that this GST is capable of transporting PAs (**Figure 5H**).

VviGST3 together with TT19 and VviGST4 are members of the Phi subgroup and clustered together with other flavonoid-related GSTs. A previous study showed that VviGST3 is unable of transport anthocyanins (Conn et al., 2008) and any ability to bind and transport others flavonoids has not been investigated. Our molecular docking analysis revealed that VviGST3 has affinity for different flavonoid ligands (**Table 1**). The highest affinity was obtained using Ep3G as ligand and a relatively high affinity was also obtained using Qu3R as ligand. Interestingly, VviGST3-flavonoid interactions indicated that the H and G sites are partly or completely overlapped when anthocyanin or flavonols were evaluated, implying that VviGST3 could not co-transport these flavonoids alongside GSH. In contrast, the H and G sites are separated when VviGST3 bind to a PA precursor, which suggest that VviGST3 could co-transport GSH (**Figure 2**). *VviGST3* was abundantly expressed in seeds with a high increase at veraison and ripening. This observation suggests that, under normal growing conditions, VviGST3 may play a key role in PA transport in seeds compared to the rest of tissues analyzed. The molecular complementation assay showed that VviGST3 was able to rescue the seed color phenotype of the *tt19-1* mutant (**Figures 5I,M**), indicating that it may function in PA transport in *Arabidopsis*. In contrast, VviGST3 was not able to restore the anthocyanin transport in *tt19-1* plants after sucrose treatment (**Figures 4** and **5D**) which is agreement with the results of Conn et al. (2008).

VviGST4 is considered an ortholog to TT19 of *Arabidopsis*; however, to the best of our knowledge, its ability to bind and transport flavonoids has only been demonstrated for anthocyanin precursors (Conn et al., 2008; Gomez et al., 2011). Taking advantage of the ability of TT19 to bind and transport anthocyanins and PAs, we compared the binding affinity of VviGST4 for flavonoids. The ΔG values of the GST-flavonoid interactions of both proteins were similar, showing affinity to four types of flavonoids (**Table 1**). These similar values for flavonoid ligands may be explained by the conservation in the amino acid residues putatively implicated in the flavonoid binding to the H-site (**Figure 1**), for instance, Leu35 and Gln40 for anthocyanins and Ala11, Leu35 and Gln40 for PAs. In this sense, Leu35 has been reported to be involved in the interaction GST-flavonoids (Kitamura et al., 2010). TT19 and VviGST4 showed a G-site located in a different place than the H-site, suggesting that these GSTs could transport anthocyanin, PAs or flavonols together with GSH. This structural characteristic was only observed for these two GSTs (**Figure 2**). The high expression of *VviGST4* in skin berries from the veraison stage correlate with the anthocyanin accumulation and is in agreement with its role as GST-anthocyanin transporter (**Figure 3**; Conn et al., 2008; Gomez et al., 2011). In addition, the low transcript levels detected in seeds may indicate that this GST could also have a function in PAs transport. This was confirmed in the complementation experiment, since *VviGST4* rescued the transparent testa phenotype of *Arabidopsis tt19-1* (**Figure 5J**). In addition, its role as GST-anthocyanin transporter was ratified with the accumulation of anthocyanins in *tt19-1* seedlings overexpressing *VviGST4* under an anthocyanins induction treatment (sucrose stress; **Figure 5E**). These results show that VviGST4 behave like *Arabidopsis* TT19 in the transport of anthocyanins and PAs, revealing a novel function of *VviGST4* in flavonoid transport.

It is tempting then to speculate that transport and accumulation of flavonoids mediated by GSTs (at least of anthocyanins and PAs) is dependent of GSH. Should this assumption be correct, the GSTs could deliver the two substrates to ABC transporters for flavonoid accumulation in the vacuole. Nonetheless, more experiments are required to test this hypothesis.

## Conclusion

In this work we propose that flavonoid transport mediated by GST in *V. vinífera* involves the participation of more than one GST in the tissues where flavonoids are accumulated. Thus, VviGST3 seem to have a predominant role in the accumulation of PAs in seeds whereas VviGST4 would act as an anthocyanin transporter in berry skin and could be transporting PAs in skin and seeds. Furthermore, high expression of *VviGST3* and *VviGST4* during late stages of fruit development also suggests their participation in other biological functions such as the formation of aroma precursors described by Kobayashi et al. (2011). Whether VviGST4 or other VviGSTs may participate in flavonol (quercetin/kaempferol) transport as is suggested by the bioinformatics predictions remains to be determined. VviGST1, on the other hand, may be involved in transporting PAs and/or flavonols in flowers and early stages of fruit development. Because of the expression pattern of VviGST1 in post-veraison seeds and its low ability to transport PAs, we postulate that VviGST3 or VviGST4 may have more relevant role in this tissue. Our work provide novel insights into flavonoids transport mediated by GSTs in grapevine and in the mechanism for the transport and accumulation of PAs in this species.

## Author Contributions

RP-D, SR-L, and EG-V conceived and designed the experiments. JM-E and JS-C performed bioinformatic experiments. RP-D and JM-E performed expression analysis and functional complementation in *Arabidopsis* tt19 mutant. RP-D,SR-L, EG-V, JM-E, and JS-C prepared the manuscript.

## Conflict of Interest Statement

The authors declare that the research was conducted in the absence of any commercial or financial relationships that could be construed as a potential conflict of interest.

## Footnotes

1 http://www.megasoftware.net

## Supplementary Material

FIGURE S1 **Phylogenetic relationship between VviGST1, VviGST3, and VviGST4 with other plant GSTs proteins**. The tree was obtained following the neighbor-joining method with 1000 replicates for boot-strap values.

FIGURE S2 **Alignment of amino acid sequences of GSTs proteins related to flavonoid transport**. The active residues for binding of GSH are indicated by a red asterisk. Hundred percentage identity is indicated with black background and about 80% identity in bold.

FIGURE S3 **Structural models of GST related to the transport of flavonoids. (A)** TT19. **(B)** VviGST1. **(C)** VviGST3. **(D)** VviGST4. Each sub-unit appears in blue and gray. In yellow the G-site (putative binding to glutathione) and in orange the H-site (putative binding flavonoids) in a monomer.

FIGURE S4 **Ramachandran plot for the modeled GSTs. (A)** TT19. **(B)** VviGST1. **(C)** VviGST3. **(D)** VviGST4. Red amino acids located in advantaged regions are presented. Yellow and coffee regions are accepted, and white regions are stereochemical not accepted.

FIGURE S5 **PROSA energy analysis of GST proteins. (A)** 5AGY: -8. **(B)** GST1: -7.55. **(C)** 4RI6: -7.55. **(D)** TT19: -6.31. **(E)** GST3: -8.21. **(F)** GST4: -6.67.

FIGURE S6 **Spatial identification of amino acids related to the putative binding of anthocyanins in TT19. (A)** In blue, amino acids suggested by Conn et al. (2008). **(B)** In red, amino acids suggested by Kitamura et al. (2012). In green, Cyanidin 3′-*O*-glucoside located in the site of lower energy.
