# Supplementary figures and images for: Differential Roles for VviGST1, VviGST3, and VviGST4 in Proanthocyanidin and Anthocyanin Transport in Vitis vinífera
Source: Front Plant Sci. 2016 Aug 3;7:1166. doi: 10.3389/fpls.2016.01166 (PMC4971086; doi:10.3389/fpls.2016.01166)

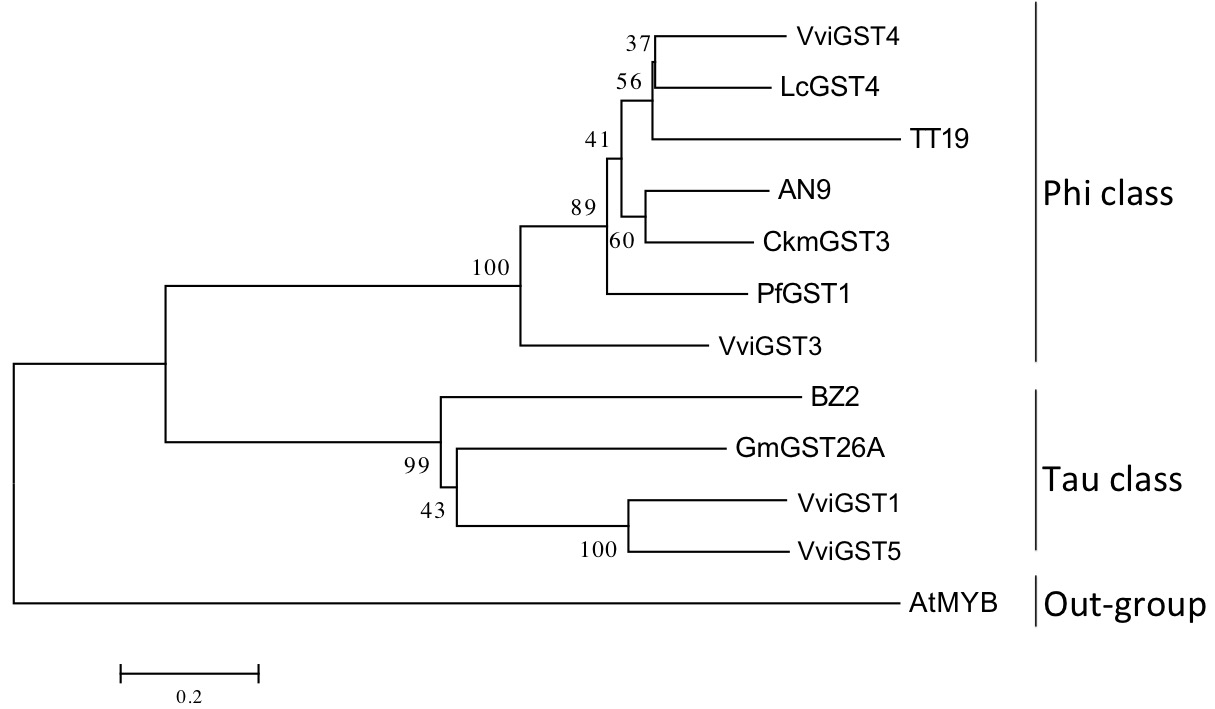

Supplement: FIGURE S1 — Phylogenetic relationship between VviGST1, VviGST3, and VviGST4 with other plant GSTs proteins. The tree was obtained following the neighbor-joining method with 1000 replicates for boot-strap values. [file Image_1.JPEG]

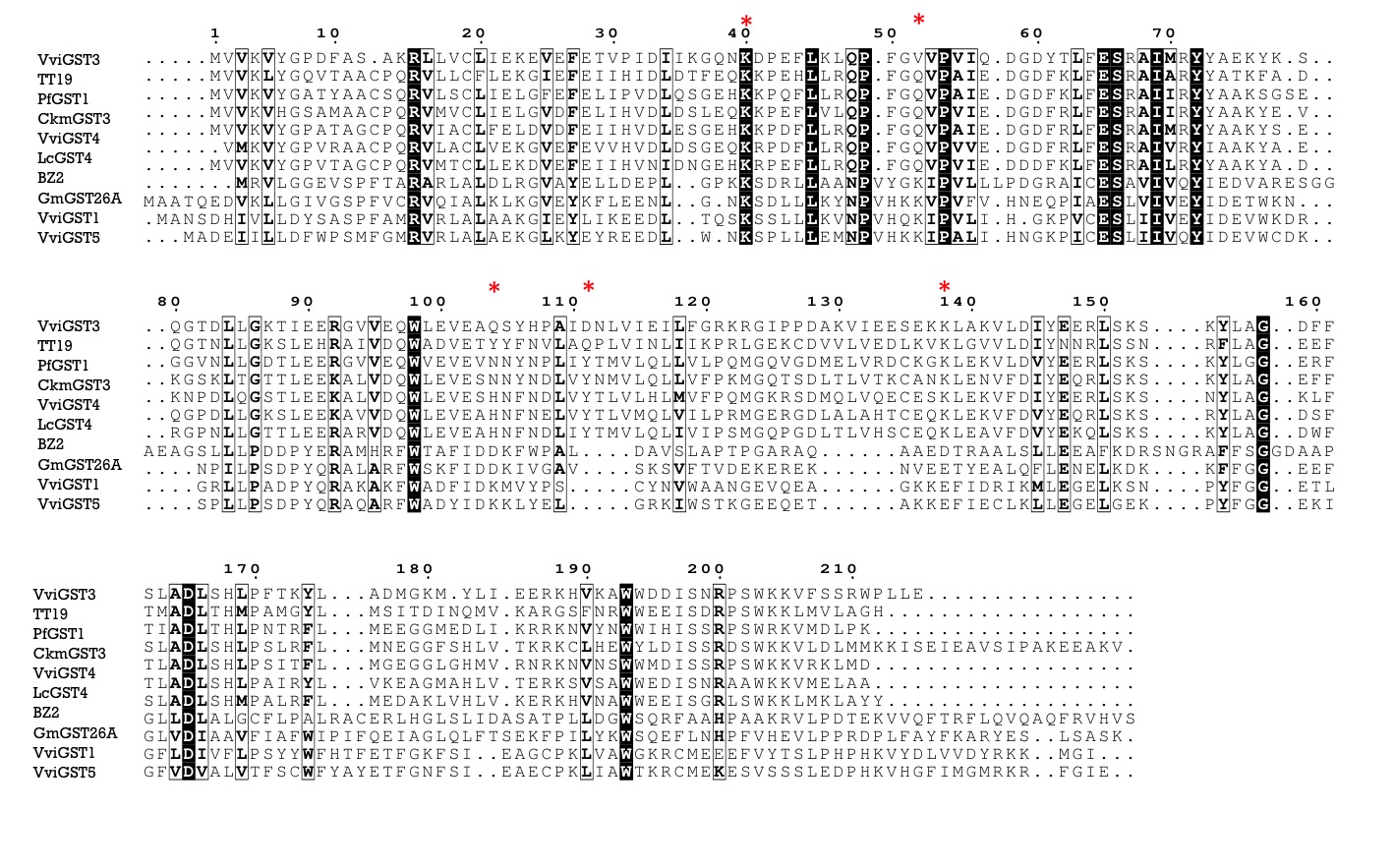

Supplement: FIGURE S2 — Alignment of amino acid sequences of GSTs proteins related to flavonoid transport. The active residues for binding of GSH are indicated by a red asterisk. Hundred percentage identity is indicated with black background and about 80% identity in bold. [file Image_2.JPEG]

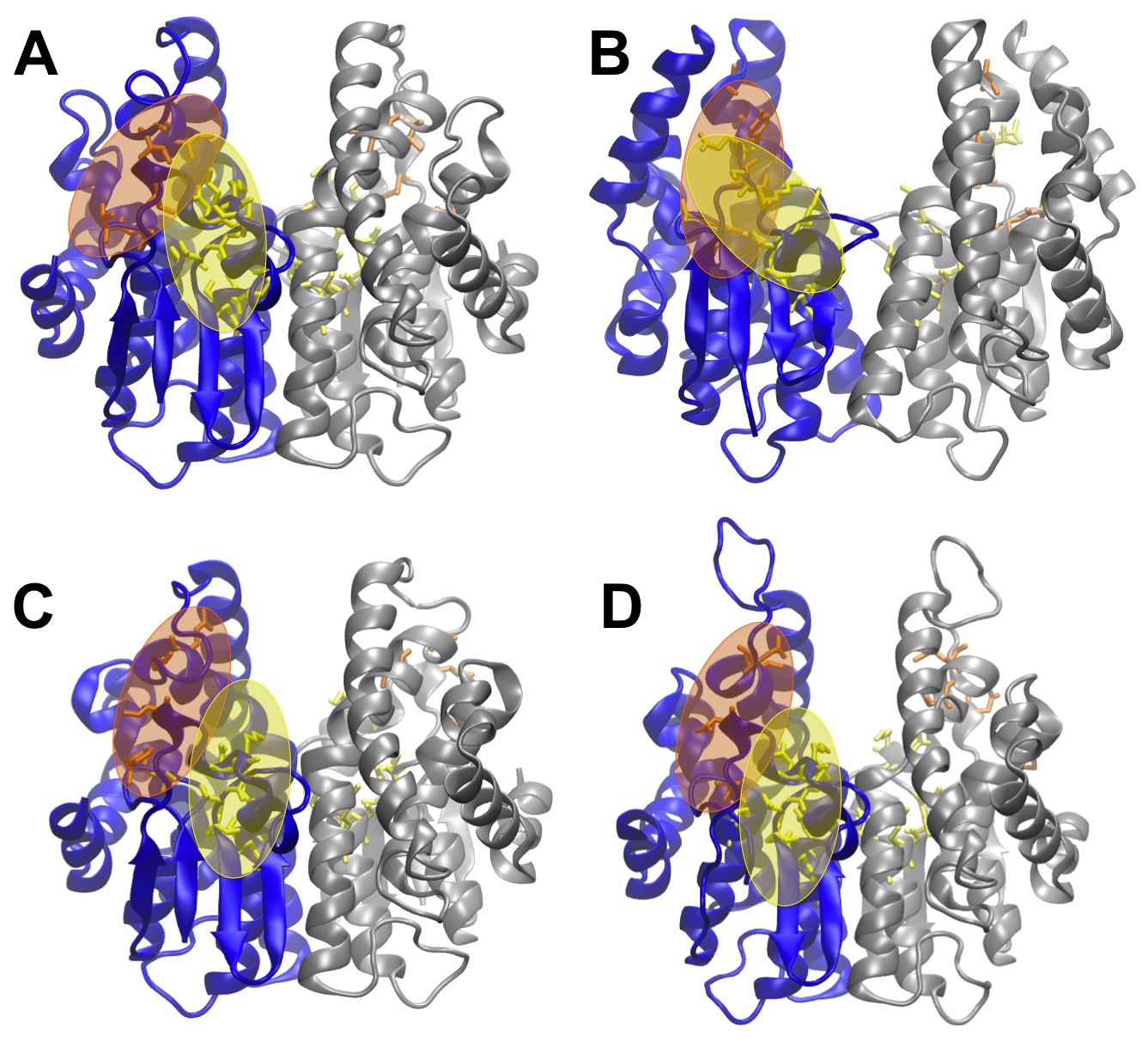

Supplement: FIGURE S3 — Structural models of GST related to the transport of flavonoids. (A) TT19. (B) VviGST1. (C) VviGST3. (D) VviGST4. Each sub-unit appears in blue and gray. In yellow the G-site (putative binding to glutathione) and in orange the H-site (putative binding flavonoids) in a monomer. [file Image_3.JPEG]

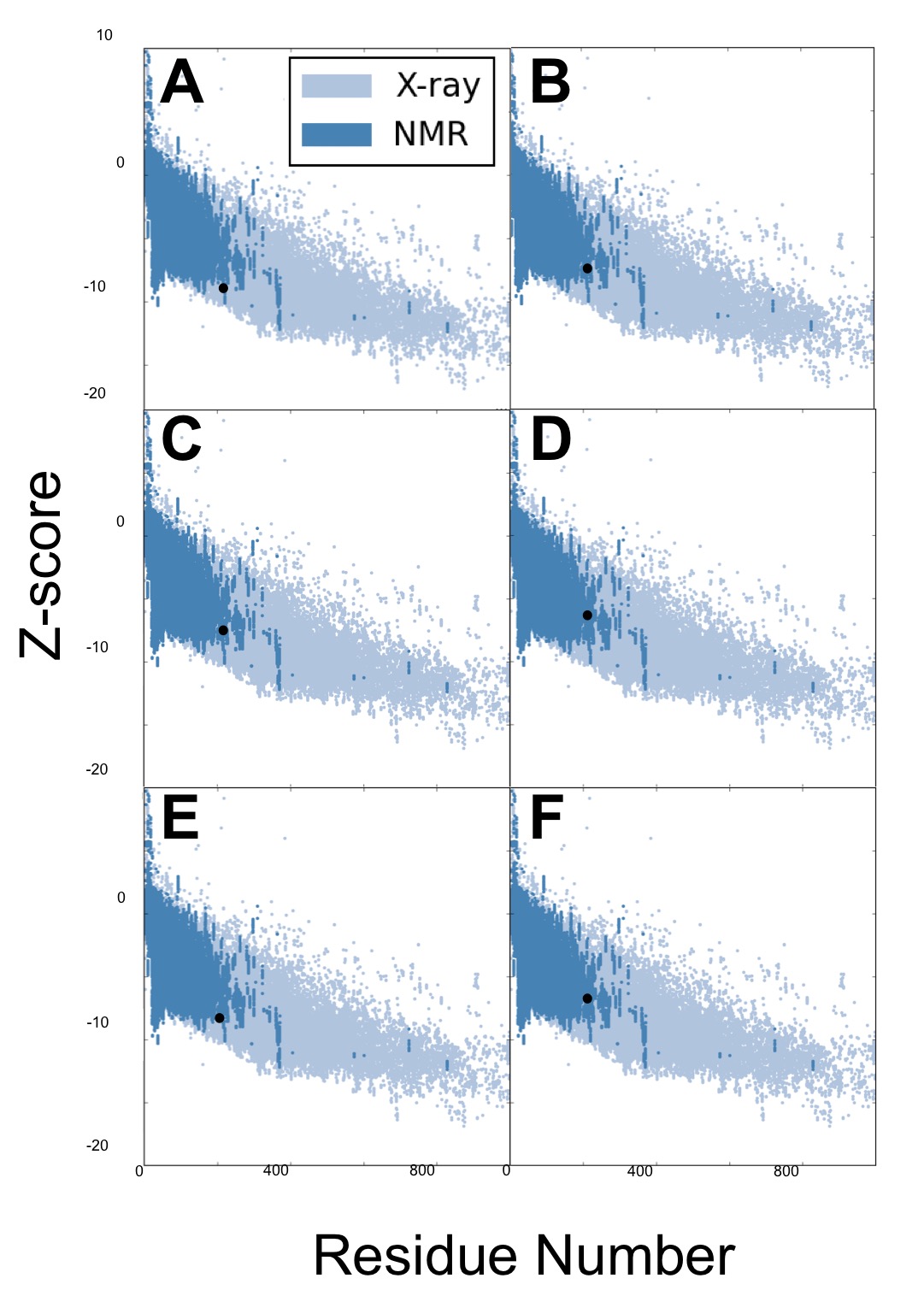

Supplement: FIGURE S4 — Ramachandran plot for the modeled GSTs. (A) TT19. (B) VviGST1. (C) VviGST3. (D) VviGST4. Red amino acids located in advantaged regions are presented. Yellow and coffee regions are accepted, and white regions are stereochemical not accepted. [file Image_4.JPEG]

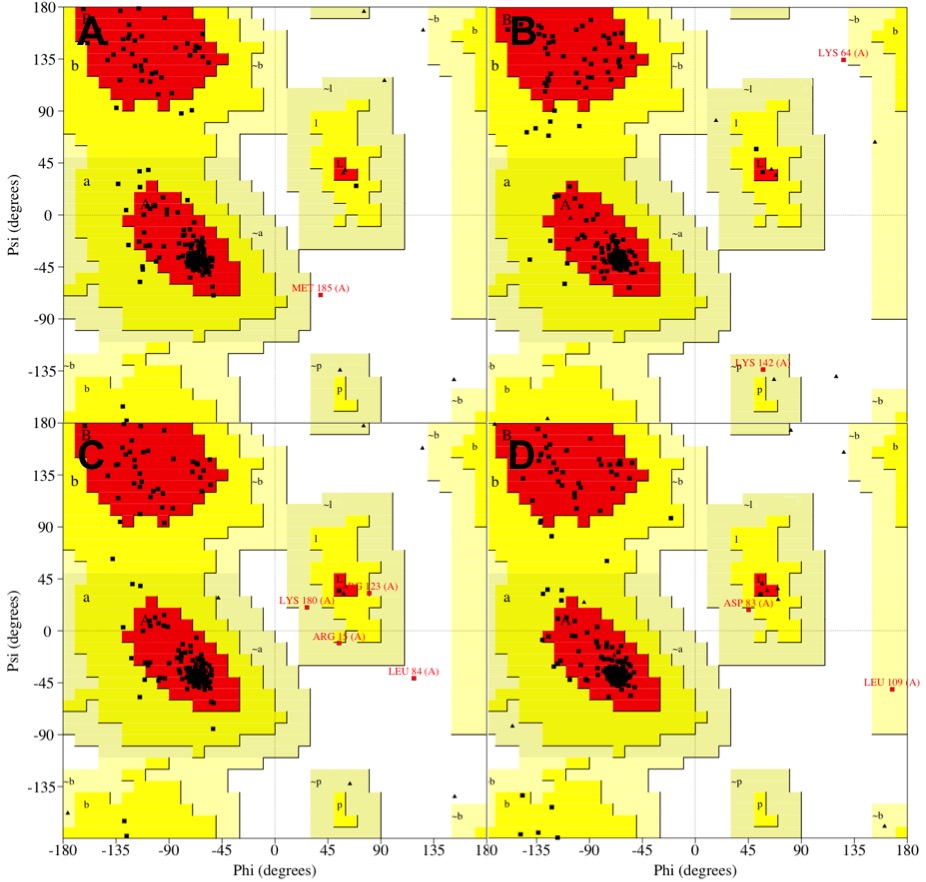

Supplement: FIGURE S5 — PROSA energy analysis of GST proteins. (A) 5AGY: -8. (B) GST1: -7.55. (C) 4RI6: -7.55. (D) TT19: -6.31. (E) GST3: -8.21. (F) GST4: -6.67. [file Image_5.JPEG]

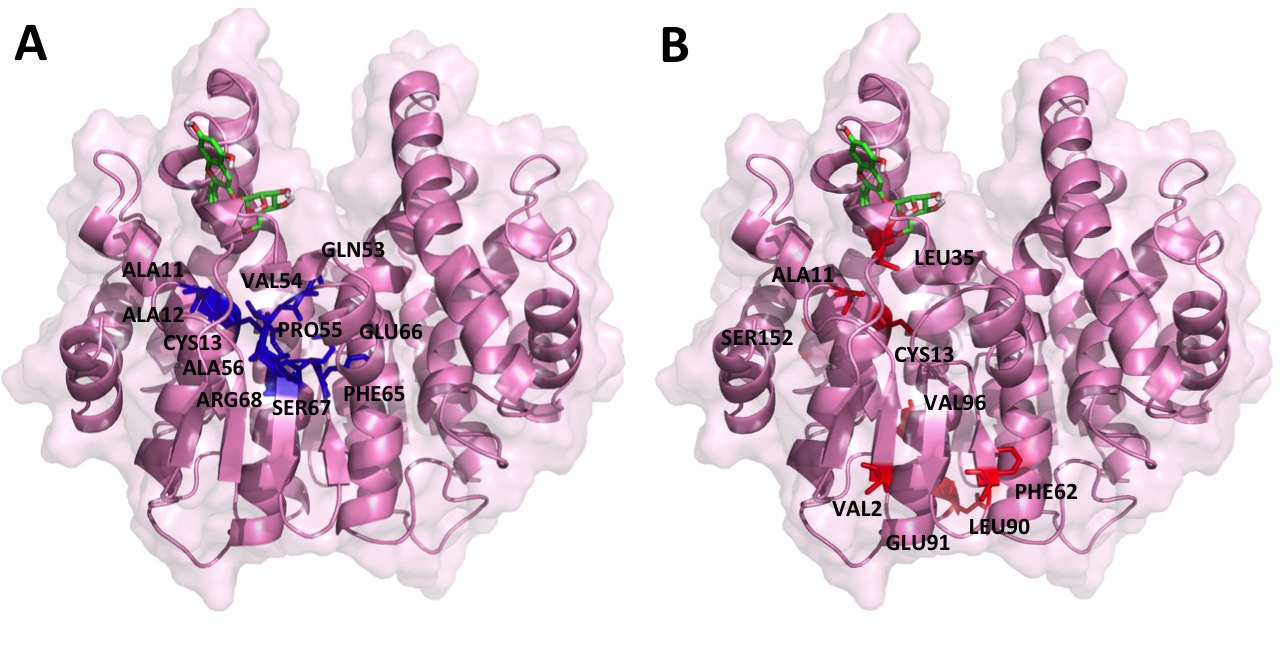

Supplement: FIGURE S6 — Spatial identification of amino acids related to the putative binding of anthocyanins in TT19. (A) In blue, amino acids suggested by Conn et al. (2008). (B) In red, amino acids suggested by Kitamura et al. (2012). In green, Cyanidin 3′-O-glucoside located in the site of lower energy. [file Image_6.JPEG]
